# From Treatment to Platform: Coupling Anaerobic Carbon Conversion with Nitrogen Removal and Phosphorus Recovery toward Circular Urban Biorefineries

**DOI:** 10.1021/acs.est.6c08923

**Published:** 2026-07-08

**Authors:** Guangze Guo, Christian Wurzbacher, Susanne Lackner, Mark C. M. van Loosdrecht, Jules B. van Lier, Jörg E. Drewes, Yujie Chen, Yu-You Li, Konrad Koch

**Affiliations:** † Chair of Urban Water Systems Engineering, 9184 Technical University of Munich, Am Coulombwall 3, 85748 Garching, Germany; ‡ Chair of Water and Environmental Biotechnology, Institute IWAR, Department of Civil and Environmental Engineering Sciences, 26536 Technical University Darmstadt, 64287 Darmstadt, Germany; § Department of Biotechnology, Delft University of Technology, Van der Maasweg 9, 2629 HZ Delft, The Netherlands; ∥ Section Sanitary Engineering, Department of Water Management, Faculty of Civil Engineering and Geosciences, Delft University of Technology, Stevinweg 1, 2628 CN Delft, The Netherlands; ⊥ Department of Civil and Environmental Engineering, Graduate School of Engineering, 13101 Tohoku University, 6-6-06 Aoba, Aramaki-Aza, Sendai, Miyagi 980-8579, Japan

**Keywords:** urban biorefineries, anaerobic membrane bioreactors, partial nitritation/anammox, phosphorus recovery

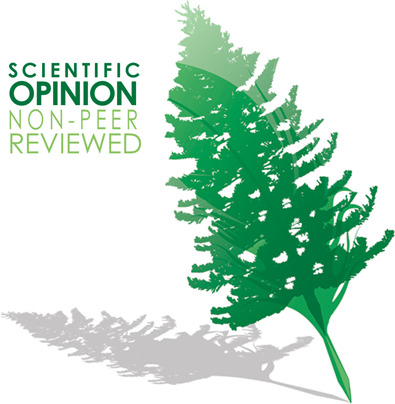

## Graphical abstract

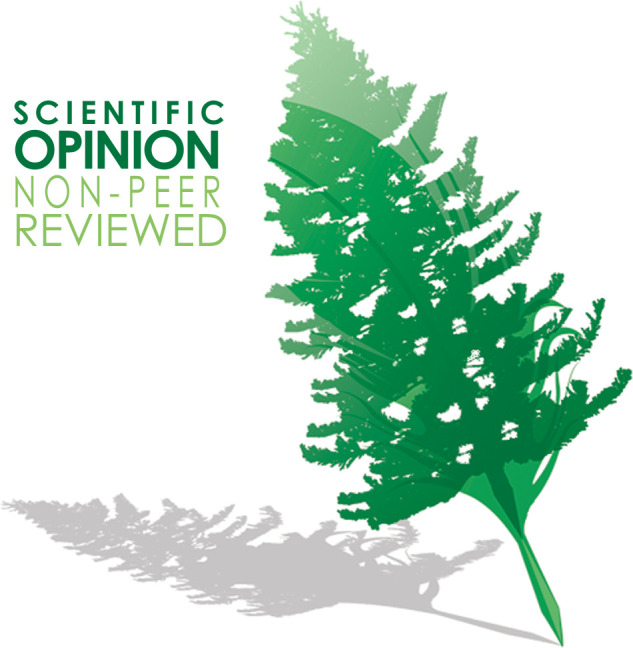

Resource recovery and nutrient management from organic waste streams are receiving more attention. Water and resource recovery facilities (WRRFs) are expected to support need-driven resource recovery at practical scale, with acceptable costs and reduced greenhouse gas (GHG) emissions. In many WRRFs, anaerobic digestion (AD) is widely implemented along the solids line for energy recovery and is frequently coupled with sidestream deammonification processes, such as partial nitritation/anammox (PN/A), to treat ammonium-rich flows from sludge dewatering.^1^ In addition, phosphorus recovery is becoming an important component of resource recovery, with physicochemical units commonly installed for phosphorus precipitation and biological oxygen demand reduction before sidestream treatment. This configuration has enabled progress in resource recovery, but it remains largely fragmented and sidestream-centric. Carbon, nitrogen, and phosphorus removal and recovery are still optimized primarily in separate process units, with limited design attention given to how the quality of the anaerobic effluent affects subsequent nitrogen removal, phosphorus recovery, and emissions control. As a result, energy costs and GHG emissions are determined by not only biological conversion but also the energy demands for the thermal hydrolysis process (THP), aeration, chemical conditioning, and sidestream operation. The central challenge is therefore no longer simply to improve isolated unit operations, but to couple upstream carbon valorization with downstream low-carbon nutrient management within a coherent treatment framework.

## AnMBRs beyond Digestion Intensification

Anaerobic membrane bioreactors (AnMBRs) combine anaerobic digestion with membrane filtration, enabling efficient methane recovery while retaining biomass and producing a low-solids permeate. AnMBRs are particularly suitable for concentrated wastewater and waste streams in which sludge granulation or gravitational settling cannot be reliably maintained and have demonstrated high energy efficiency and strong resource recovery potential.^2^ In contrast to this sidestream-oriented processing, AnMBRs should not be regarded simply as a digester upgrade or as another process intensification strategy, such as vacuum-assisted fermentation, THP, or plug-flow digestion, but as a membrane-governed configuration that decouples hydraulic retention time (HRT) from solids retention time (SRT), thereby supporting prolonged hydrolysis and stable methanogenic activity at high solids concentrations. Vacuum-assisted fermentation also represents an alternative route for decoupling HRT and SRT; however, AnMBRs further govern hydraulic discharge and permeate quality for downstream nutrient management.^3^ Although volumetric gains for sludge digestion may be modest, extended solids retention may still enhance carbon conversion and reduce reliance on thermal hydrolysis. More importantly, membranes define the liquid stream presented to downstream nutrient management processes.

Nevertheless, even under optimized operation, incomplete anaerobic conversion implies that a fraction of biodegradable organic matter is discharged from the reactor without being fully digested. As demonstrated in full-scale WWTPs, residual methane formation during downstream sludge buffering and storage represents a system-level challenge of anaerobic sludge processing,^4^ which can be managed through (1) improving digestion efficiency or intensified anaerobic configurations to reduce the residual methane potential, (2) recycling methane-containing ventilation gas, for example, as combustion air in gas engines, and (3) oxidizing highly diluted methane streams rather than ventilation-based dilution and release. The main scale-up challenge is therefore whether membrane-governed high-solids operation can be sustained with acceptable energy demand and operating expenditure, which ultimately determines the techno-economic feasibility of AnMBRs. Instrumentation, control, and automation (ICA) systems are key to maintaining stable operation by regulating flux, transmembrane pressure, and solids concentration.

## Coupling Nitrogen Removal with Phosphorus Mineralization

Full-scale PN/A has developed from pilot demonstrations to a well-established sidestream option. PN/A offers lower aeration demand and reduced external carbon requirements than conventional nitrification–denitrification, but stable operation depends on retaining slow-growing anammox biomass while avoiding nitrite accumulation. Application surveys show that practical constraints include (1) sensitivity to influent solids and the associated loss of active biomass, (2) operational problems related to biomass retention, settling, and solids separation, and (3) the risk of N_2_O emissions.^5^ In the proposed “AnMBR+” configuration, an upfront AnMBR fully retains particulate material, eliminating the stress of solids on the downstream PN/A. Hydroxyapatite (HAP)-enhanced PN/A further supports biomass retention by promoting calcium–phosphate mineral formation in granules. Ca–P enrichment increases PN/A granule density and settleability, helping to maintain the stratification of ammonia-oxidizing and anammox biomass. A phosphorus removal rate (PRR) to nitrogen removal rate (NRR) ratio of ≥0.02 reflects a kinetic balance between HAP formation and microbial growth, enabling stable mineral-aided granulation.^6^ This configuration integrates nitrogen removal with in situ mineral formation in a single reactor, avoiding an additional separation step between the anaerobic stage and downstream nitrogen treatment.

Compared with struvite-based phosphorus recovery, HAP-PN/A yields mechanically more stable granules and better aligns with the ionic composition of many wastewater streams, where calcium is typically more abundant than magnesium. However, calcium availability in digestate may vary depending on feedstock composition and upstream processes. Still, controlling N_2_O emissions while maintaining stable operation remains a key priority. Full-scale evidence indicates that N_2_O emissions originate predominantly from the nitritation step and are closely linked to nitrite accumulation under low-dissolved oxygen conditions. Accordingly, in one-stage HAP-PN/A systems where nitritation cannot be independently regulated, N_2_O mitigation relies on limiting nitrite accumulation and promoting rapid nitrite consumption within stratified biomass structures.

## System-Level Trade-offs in Energy, Emissions, and Recovery

For AnMBR-based systems, methane recovery alone is unlikely to determine economic viability; membrane operation, post-treatment requirements, and recovered-product value must also be considered under practical application scenarios. When integrated into a unified platform, AnMBR–HAP–PN/A systems exhibit different life-cycle assessment (LCA)/techno-economic analysis (TEA) sensitivities compared to THP–AD-based sidestream configurations. Methane recovery from AnMBRs can offset fossil-derived energy use; however, net energy and GHG emission reduction depend on membrane-related energy demand and the management of N_2_O emissions associated with PN/A operation. Calcium salts and alkalinity control can add embodied carbon, although in situ HAP formation may reduce reliance on separate struvite-based phosphorus recovery units. The evaluation should therefore shift from removal efficiency alone toward resource recovery opportunities, operating inputs, and avoided environmental burdens. Accordingly, performance metrics expressed per unit of recovered resource, such as kWh_e_ kg_VS_^–1^ converted or € kg_P_^–1^ recovered, are more meaningful than removal-based indicators alone. The platform improves carbon retention and enables phosphorus recovery, whereas nitrogen is primarily removed rather than recovered. Direct nitrogen recovery would require alternative routes, such as electrochemical ammonium extraction after membrane filtration.^7^ Recent system-level TEAs suggest that integrating energy recovery with phosphorus recovery can improve the economic balance of treatment platforms, although outcomes depend strongly on sludge disposal costs and nutrient backload management.^8^ Retrofitting existing facilities rather than constructing new installations could further reduce capital expenditures and align well with typical utility investment cycles. Therefore, LCA/TEA should be integrated early in process design rather than applied only after implementation, although results will remain dependent on system boundaries and assumptions.

## Implications

The AnMBR–HAP–PN/A configuration is better viewed as a case-dependent option rather than a universal upgrade to existing treatment systems. Its practical relevance depends on whether carbon conversion, nitrogen removal, and phosphorus mineralization can be jointly governed within acceptable limits of energy demand, emissions, and operational complexity. From this perspective, three validation criteria are central. First, membrane-governed high-solids operation needs to be sustained over long time scales at acceptable energy demand, supported by effective cleaning strategies. Second, PN/A requires conditions that avoid sustained nitrite accumulation and limit N_2_O emissions under realistic loading disturbances. Third, system-level TEA/LCA should be conducted under site-specific conditions and expressed per unit of recovered resource. The value of such configurations lies not in combining advanced units, but in enabling more designable and predictable coupling between upstream carbon recovery and downstream nutrient management. The integrated platform offers a credible alternative to established AD-based sidestream recovery schemes when these conditions are met (Figure 1).

**Figure 1:**
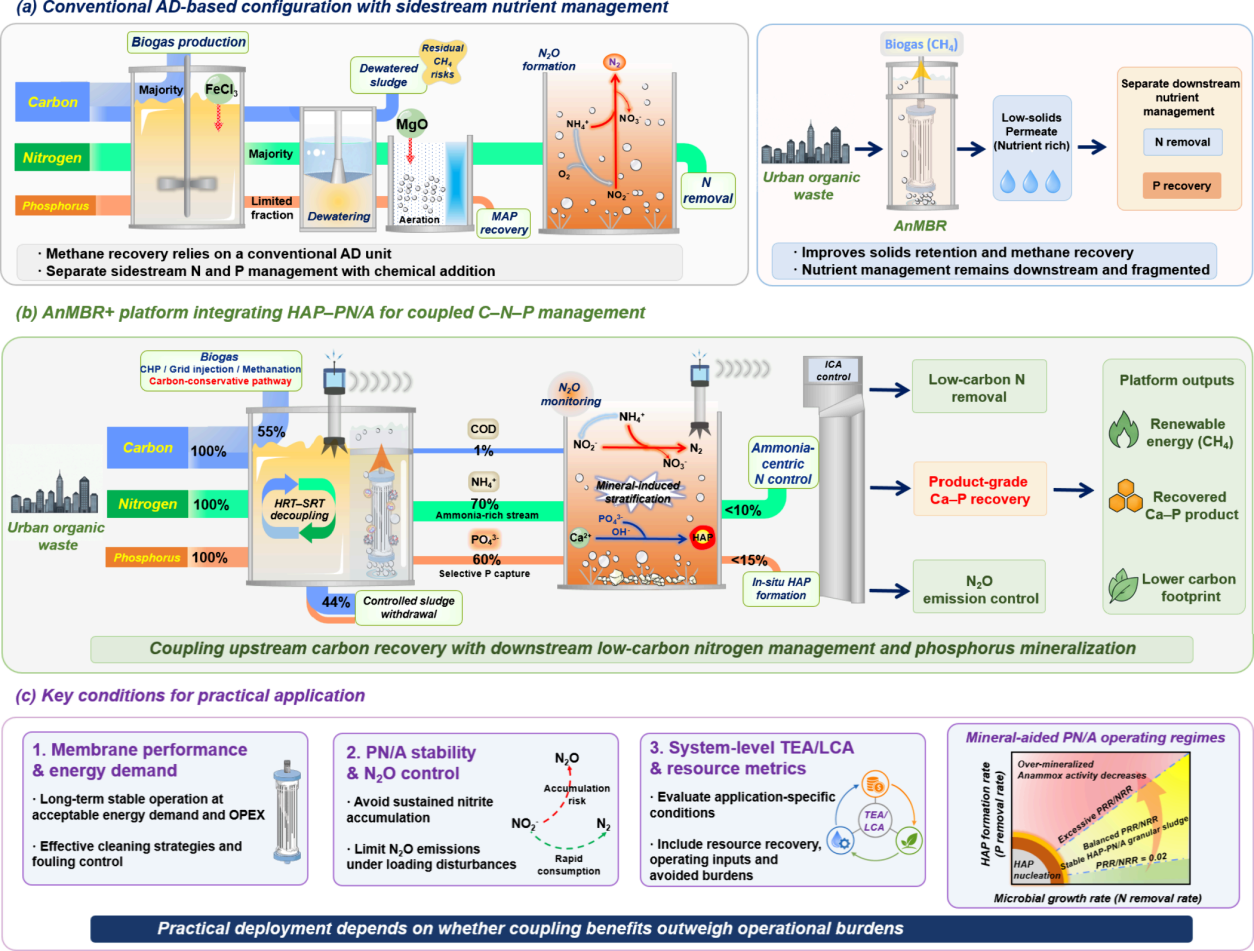
System-level comparison of (a) conventional AD-based sidestream management and (b) an integrated AnMBR–HAP–PN/A configuration, highlighting the design logic for coupling upstream carbon recovery with downstream low-carbon nitrogen management. (c) Several key challenges for the practical implementation remain, which are highly side-specific.
